# Database resources of the National Genomics Data Center, China National Center for Bioinformation in 2026

**DOI:** 10.1093/nar/gkaf1172

**Published:** 2025-12-08

**Authors:** CNCB–NGDC Members and Partners, Yiming Bao, Zhang Zhang, Wenming Zhao, Jingfa Xiao, Shuhui Song, Shunmin He, Guoqing Zhang, Yixue Li, Guoping Zhao, Runsheng Chen, Yibo Wang, Weijie Zhang, Xiaoning Chen, Yanling Sun, Bixia Tang, Yu Zhang, Kai Liu, Bing Su, Yaoxi He, Tingrui Song, Yirong Shi, Yanyan Li, Di Hao, Kaixin Zhan, Tao Xu, Wenyan Lei, Cuidan Li, Hengyu Zhou, Zhi Nie, Anke Wang, Pan Li, Peihan Wang, Zhuojing Fan, Rongxi Zhu, Haoyu Cheng, Yuxian Guo, Liya Yue, Xiaoyuan Jiang, Renjun Gao, Yongjie Sheng, Haitao Niu, Tuohetaerbaike Bahetibieke, Wenbao Zhang, Fei Chen, Jiayue Meng, Mengyao Han, Yuwei Huang, Liyun Yuan, RuiKun Xue, Jingyao Zeng, Xini Meng, Qifei Wang, Yiwen Hu, Yulan Deng, Na Ai, Zheng Huang, Yun Li, Yang Yuan, Guochao Li, Lan Jiang, Ting Li, Ling Zuo, Jianxin Chen, Qianqian Peng, Sijia Wang, Sicheng Wu, Hao Jiang, Hailong Kang, Jiawei Shi, Nianguo Dong, Ximiao He, Fei Yang, Shuai Jiang, Zhenxian Han, Xue Bai, Dong Zou, Sisi Zhang, Yi Wang, Zhijian Duan, Lun Li, Wenjing Sun, Sijia Zhang, Quan Luo, Jinying Han, Hairong Huang, Adong Shen, Jing Wang, Hao Wen, Congfan Bu, Xiaotong Ji, Qiheng Qian, Hao Zhang, Qingyun Cai, Kaiwen Zhang, Xiaoqing Jiang, Mingkun Li, Hong Luo, Zishan Wu, Siwei Ren, Haixia Xie, Zhixiang Yuan, Dongmei Tian, Demian Kong, Shaoqi Bei, Yueyue Wu, Lei Liu, Suqi Cao, Weili Lin, Ruixin Zhu, Dingfeng Wu, Yuyan Meng, Wan Liu, Pingping Wang, Xinhao Zhuang, Xing Yan, Zhihua Zhou, Wenxing Gao, Qiang Li, Na Jiao, Yiyun Liu, Lili Tian, Wei Zhao, Wenting Zong, Xinchang Zheng, Xu Chen, Tingting Chen, Xiaolong Zhang, Yubo Zhou, Junwei Zhu, Bing Xu, Lili Dong, Caixia Yu, Wenjie Li, Shuang Zhai, Yubin Sun, Qiancheng Chen, Yanqing Wang, Xuetong Zhao, Shaosen Zhang, Jinbiao Wang, Yuhao Zeng, Zheng Luo, Yiran Zhan, Zihan Wang, Xi Zhao, Yuxi Liu, Lina Ma, Yingke Ma, Meili Chen, Tongtong Zhu, Wenzhuo Cheng, Yuan Chu, Ming Chen, Tianyi Xu, Hao Gao, Si Zheng, Jialin Mai, Jinbei Wang, Rui Tang, Jiao Li, Yue Qi, Zhao Li, Zhuang Xiong, Xupeng Chen, Yaoke Wei, Xiangyu Yu, Rujiao Li, Mochen Zhang, Huiying Chen, Guoliang Wang, Song Wu, Hongzhu Qu, Xiangdong Fang, Enhui Jin, Dongli Zhao, Gangao Wu, Zhonghuang Wang, Zhiyao Wei, Zhe Zhang, Yuanguang Meng, Yongrong Cao, Xuemei Lu, Yanan Wang, Wei Liu, Jinyan Huang, Yanfang Jiang, Guoyue Lv, Xinyu Liu, Guowang Xu, Xumin Wang, JiangYong Qu, Baisheng Li, Chang Zhang, Xiaofeng Zou, Guoxi Zhang, You Guo, Weiwei Jin, Jing Gong, Xiaohui Niu, Wenkang Shen, Anyuan Guo, Zhixiang Zuo, Jian Ren, Xinxin Zhang, Yun Xiao, Xia Li, Dan Liu, Yu Xue, Zheng Zhao, Tao Jiang, Wanying Wu, Fangqing Zhao, Jinyang Zhang, Xianwen Meng, Ming Chen, Bowen Song, Jia Meng, Yujie Gou, Miaomiao Chen, Di Peng, Hao Luo, Feng Gao, Jie Jiang, Kunqi Chen, Xinhe Huang, Chi Zhang, Chunjie Liu, Guiyan Xie, Hao Yuan, Tianhan Su, Yong E Zhang, Chenfen Zhou, Yincong Zhou, Guoji Guo, Qiong Zhang, Shanshan Fu, Miaoying Zhao, Tong Chen, Yuan Yuan, Dachao Tang, Ming Lei, Mei Luo, Yubin Xie, Yaru Miao, Jiongming Ma, Haokai Ye, Bowen Song, Daiyun Huang, Yuxin Zhang, Di Zhang, Jianzhen Peng, Xingyu Liao, Xin Gao, Jianxin Wang, Jiang Li, Chunhui Yuan, Dechang Yang, Feng Tian, Ge Gao, Wenyi Wu, Cheng Han, Juntian Qi, Ni A An, Chuan-Yun Li, Xuan Wang, Zhen Wei, XiaoTong Luo, Jiaxing Yue, Zepu Miao, Qing Tang, Zihao Feng, Bo Liu, Jian Yang, Chenyu Yang, Leming Xiao

## Abstract

The National Genomics Data Center (NGDC), as part of the China National Center for Bioinformation (CNCB), provides a suite of database resources for worldwide researchers. As multi-omics big data and artificial intelligence reshape the paradigm of biology research, CNCB–NGDC continuously updates its database resources to enhance data usability, foster knowledge discovery, and support data-driven innovative research. Over the past year, notable progress has been achieved in expanding the scope of high-quality multi-omics datasets, building new database resources, and optimizing extant core resources. Notably, the launch of BIG Search enables cross-database search services for large-scale biological data platforms, including NGDC, National Center for Biotechnology Information (NCBI), and European Bioinformatics Institute (EBI). Additionally, several new resources have been developed, covering genome and variation (Hiland Resource, TOAnnoPriDB), expression (TEDD), single-cell omics (PreDigs, scMultiModalMap, TE-SCALE), radiomics (TonguExpert), health and disease (CAVDdb, IDP, MTB-KB, ResMicroDb), biodiversity and biosynthesis (SugarcaneOmics), as well as research tools (Dingent, miMatch, OmniExtract, RDBSB, xMarkerFinder). All these resources and services are freely accessible at https://ngdc.cncb.ac.cn.

## Graphical Abstract

**Graphical Abstract:**
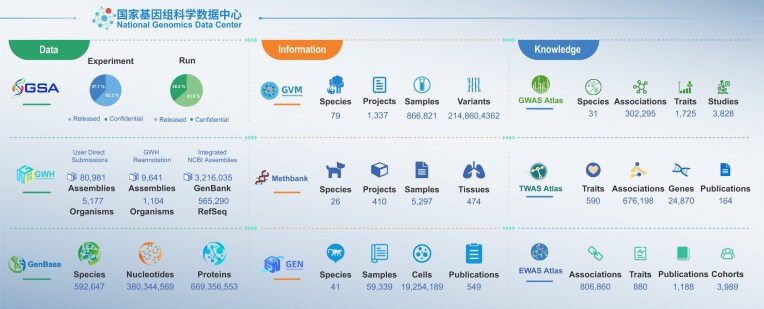

## Introduction

The National Genomics Data Center (NGDC), established in 2019, is administratively affiliated with the China National Center for Bioinformation (CNCB), Beijing Institute of Genomics (BIG), and Chinese Academy of Sciences (CAS) [1]. In collaboration with the Institute of Biophysics and the Shanghai Institute of Nutrition and Health of CAS, CNCB–NGDC has established strategic partnerships with numerous organizations nationwide (https://ngdc.cncb.ac.cn/partners).

Rapid advances in high-throughput sequencing are propelling biology into a multi-omics era, with single-cell and spatial omics techniques further increasing data dimensionality and resolution [2–4]. Worldwide large-scale initiatives, such as All of Us [5], Our Future Health [6], Human Cell Atlas [7], Earth BioGenome Project [8], Single-Cell Expression Atlas [9], UK Biobank [10], and ImmPort [11], have generated extensive multimodal datasets across diverse species, tissues, and populations. In parallel, burgeoning repositories of biomedical images and image-derived phenotypes have emerged. Together, these resources provide unprecedented detail for system-level characterization—from cellular atlases and interactions to immune microenvironments, as well as organ- and tissue-level morphology—thereby enabling a wide range of studies including developmental processes [12–14], immune responses [15–17], aging mechanisms [18, 19], disease etiology [20–22], and potential therapeutic targets [23–25]. At the same time, artificial intelligence (AI) is catalyzing a paradigm shift [26], with landmark models such as AlphaFold [27], Geneformer [28], and scGPT [29]. This, in turn, demands standardized, interoperable, and reusable (“AI-ready”) data foundations and rigorous benchmarking systems [30].

**Figure 1.**
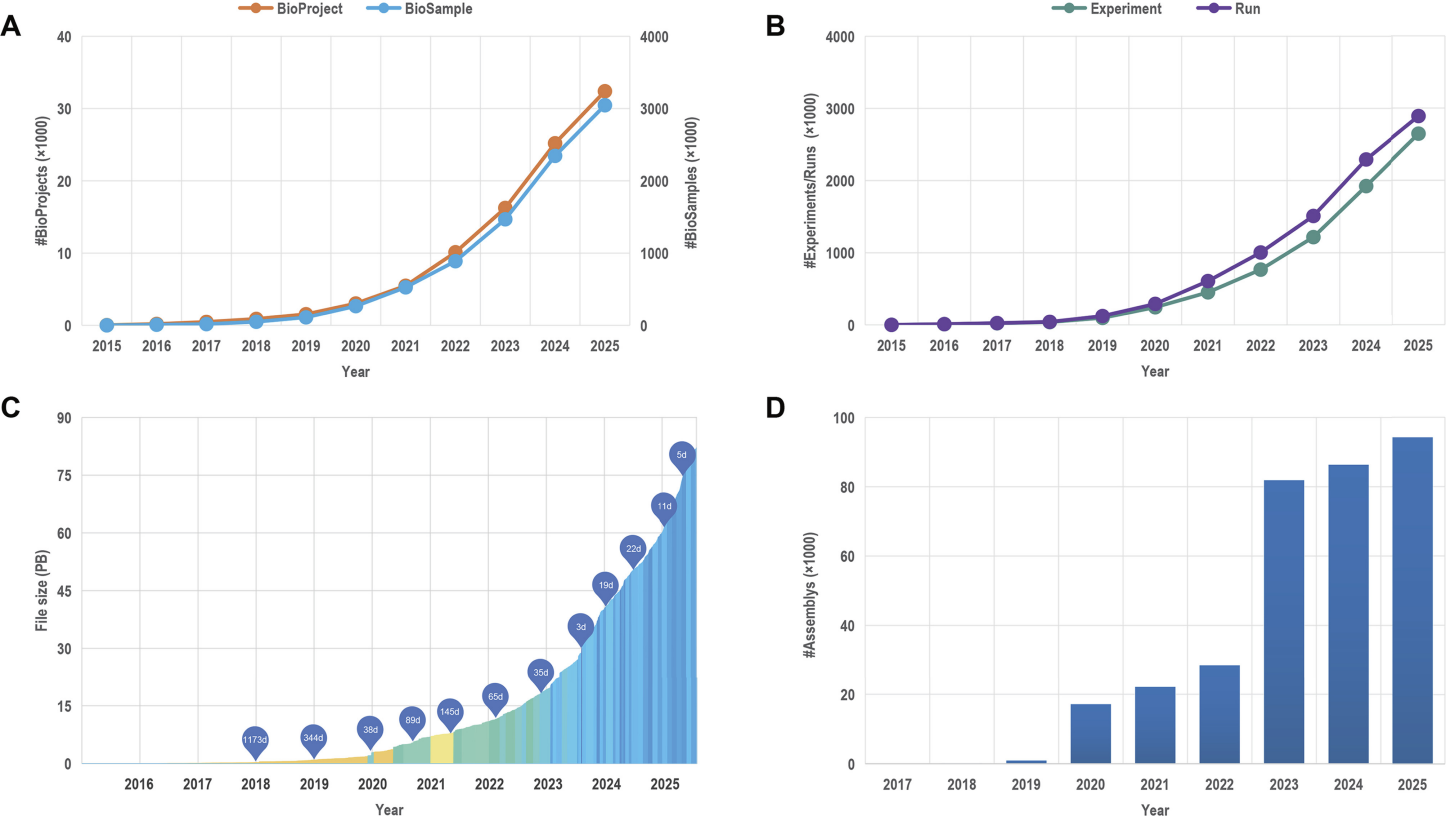
Overview of data submissions to CNCB–NGDC. (**A**) Statistics for BioProject and BioSample. (**B**) Statistics for Experiments and Runs in GSA. (**C**) Timeline of data growth in GSA. (**D**) Statistics for genome assemblies in GWH. All statistics are regularly updated and publicly accessible at https://ngdc.cncb.ac.cn/bioproject, https://ngdc.cncb.ac.cn/biosample, https://ngdc.cncb.ac.cn/gsa, and https://ngdc.cncb.ac.cn/gwh.

**Figure 2.**
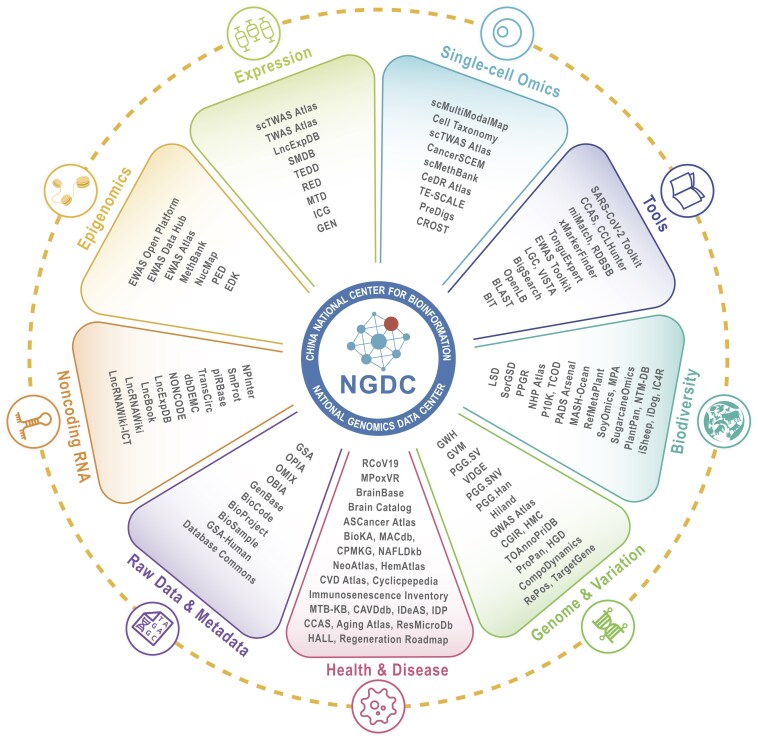
The core database resources of CNCB–NGDC organized into major categories. These resources are publicly accessible and searchable via the CNCB–NGDC homepage (https://ngdc.cncb.ac.cn). A full list of databases is available at https://ngdc.cncb.ac.cn/databases.

**Figure 3.**
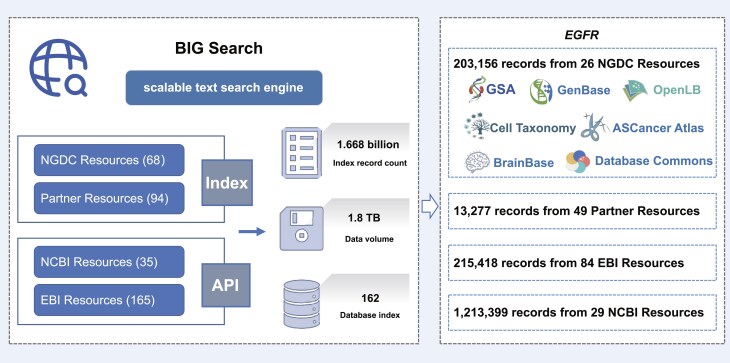
BIG Search, developed by CNCB–NGDC, integrates heterogeneous data resources from global data centers, enabling scalable and cross-domain text retrieval.

Within this context, over the past year, CNCB–NGDC has further expanded its network of subcenters throughout the country (https://ngdc.cncb.ac.cn/subcenter), covering a diversity of data resources related to biodiversity, medicine, tumor, pathogen, marine, etc. (Table 1). Based on this, CNCB–NGDC has launched several new resources and continuously updated existing ones, committed to providing an increasingly comprehensive and intelligent suite of biological resources (Fig. 1) [31–39]. These data resources, knowledge information, and analytical tools span multiple omics fields, including genome, transcriptome, epigenome, and radiome, and are widely applied in research areas such as precision medicine, embryonic development, immune responses, and aging mechanisms. These core resources are further interlinked, forming an integrated network that facilitates seamless traversal across databases, retrieval of contextually pertinent information, and comprehensive inquiry (Fig. 2). In addition, NGDC has significantly enhanced its cross-platform search capabilities, integrating data from important biological resource platforms such as NCBI and EBI (Fig. 3). This has further facilitated the efficient aggregation and coordinated management of heterogeneous, multi-source datasets, improving data utilization efficiency and accessibility, and ensuring that global researchers can effectively integrate and utilize biological data resources across platforms and databases. CNCB–NGDC adheres to its core mission of providing biological information resources and data analysis services to the global research community, committed to supporting researchers at all levels. Broad biological researchers have access to foundational information on biological literature, codes, and databases, enabling a deeper understanding of the field of bioinformatics. Meanwhile, specialist researchers are provided with high-quality data resources that support the rapid iteration of AI models, facilitating their application in specific research domains. Here, we summarize recent developments at CNCB–NGDC and present its core resources and services (Table 2). All resources and services are publicly accessible through the CNCB–NGDC homepage (https://ngdc.cncb.ac.cn).

**Table 1.** The NGDC subcenters network

Subcenter name	Abbreviation	Affiliation	Location	Research focus	Joined date
Biodiversity	NGDC-BDV	Kunming Institute of Zoology, Chinese Academy of Sciences	Kunming	Advancing biodiversity data across ecological, species, and genetic levels	December, 2023
Tumor gene diagnosis data	NGDC-TGD	The Biomedical Big Data Center, The First Affiliated Hospital, Zhejiang University School of Medicine	Hangzhou	Managing tumor genetic data to enhance cancer diagnostics and clinical outcomes	April, 2024
Traditional Chinese Medicine	NGDC-TCM	China Academy of Chinese Medical Sciences	Beijing	Standardizing TCM data resources by integrating proteomic, metabolomic, and transcriptomic information	June, 2024
Pathogenic microorganism	NGDC-PMO	Guangdong Provincial Center for Disease Control and Prevention	Guangzhou	Sharing pathogenic microorganism genomic data to improve accessibility	August, 2024
Northeast medical genomics	NGDC-NMG	The First Hospital of Jilin University	Changchun	Building region-specific genomic databases with integrated clinical and multi-omics data	November, 2024
Marine organism genomics	NGDC-MOG	Yantai University	Yantai	Developing marine genomic data systems for blue economy and ecological protection	December, 2024
Metabolomics	NGDC-MET	Dalian Institute of Chemical Physics, Chinese Academy of Sciences	Dalian	Enabling standardized and intelligent analysis of large-scale metabolomics data	May, 2025
Gannan medical genomics	NGDC-GMG	The First Affiliated Hospital of Gannan Medical University and The First People’s Hospital of Nankang District	Ganzhou	Establishing a precision medicine ecosystem in Ganzhou through genomic data and biospecimen integration	May, 2025

**Table 2.** The recent updated database resources of CNCB–NGDC

Resource type	Resource name	URL	Short description	Records	Growth	Audience
Search	BIG Search	*https://ngdc.cncb.ac.cn/search*	A cross-database search platform for biological resources	1 699 078 390	15.44%	General audience
Knowledge	Database Commons	*https://ngdc.cncb.ac.cn/databasecommons*	A catalog of worldwide biological databases	7346	6.19%	General audience
Knowledge	BioCode	*https://ngdc.cncb.ac.cn/biocode*	A centralized repository dedicated to archiving bioinformatics tool codes	7536	38.62%	General audience
Knowledge	OpenLB	*https://ngdc.cncb.ac.cn/openlb/home*	An open-access platform for searching bioscience literature texts	39 880 128	5.71%	General audience
Data—raw data and metadata	BioProject	*https://ngdc.cncb.ac.cn/bioproject*	A public repository of biological research projects	32 394	55.49%	General audience
Data—raw data and metadata	BioSample	*https://ngdc.cncb.ac.cn/biosample*	A public repository of biological research samples	3 046 071	52.19%	General audience
Data—raw data and metadata	GSA	*https://ngdc.cncb.ac.cn/gsa*	An open-access repository for non-human raw sequence reads	2 457 662	52.13%	General audience
Data—raw data and metadata	GSA-Human	*https://ngdc.cncb.ac.cn/gsa-human*	A GSA sub-database specialized for human genetic omics data	4 366 361	27.88%	General audience
Data—raw data and metadata	OMIX	*https://ngdc.cncb.ac.cn/omix*	A public repository for submitting and sharing diverse life-science data	57 883	160.70%	General audience
Data—raw data and metadata	GenBase	*https://ngdc.cncb.ac.cn/genbase*	An open-access repository for nucleotide sequence archiving and sharing	1 114 470	17.93%	General audience
Data—genome	Genome Warehouse	*https://ngdc.cncb.ac.cn/gwh*	A vital repository for whole-genome sequencing and annotation data	94 204	11.81%	Genomics researcher
Data—genome	Hiland Resource	*https://ngdc.cncb.ac.cn/hiland/*	A database integrating phenome, genome, and GWAS data of highland populations	700 342	–	Highland population genomics researcher
Data—genome	SugarcaneOmics	*https://ngdc.cncb.ac.cn/scod/*	A comprehensive platform for sugarcane functional genomics and breeding applications	14	–	Sugarcane genomics researcher
Data—variation	GVM	*https://ngdc.cncb.ac.cn/gvm*	A global repository of genomic sequence variations across all species	∼2.09 billion	2.96%	Genomic variation researcher
Data—variation	TOAnnoPriDB	*http://bigdata.ibp.ac.cn/TOAnnoPriDB*	An integrative database for trans-omic annotations and variant prioritization	∼220 million	–	Non-coding variant researcher
Data—transcriptome	Gene Expression Nebulas	*https://ngdc.cncb.ac.cn/gen*	A database of transcriptomic profiles analyzed through a unified pipeline	86 584	44.87%	Transcriptomics researcher
Data—transcriptome	LncExpDB	*https://ngdc.cncb.ac.cn/lncexpdb*	A repository of human long non-coding RNA expression profiles	39 253	55.8%	Non-coding rna researcher
Data—transcriptome	CROST	*https://ngdc.cncb.ac.cn/crost*	A platform integrating data, knowledge, and tools for spatial transcriptomics	1313	30.99%	Spatial transcriptomics researcher
Data—transcriptome	TWAS Atlas	*https://ngdc.cncb.ac.cn/twas*	A comprehensive knowledgebase of transcriptome-wide association studies	274 972	68.53%	Human TWAS researcher
Data—transcriptome	TEDD	*https://ngdc.cncb.ac.cn/tedd*	A resource for the dynamics of human translation efficiency (TE, TR, EVI)	1518	–	Translation efficiency researcher
Data—epigenome	EWAS Open Platform	*https://ngdc.cncb.ac.cn/ewas*	A comprehensive resource for epigenome-wide association studies	898 918	85.15%	Human EWAS researcher
Data—epigenome	MethBank	*https://ngdc.cncb.ac.cn/methbank*	A comprehensive DNA methylation database across various species	5297	49.08%	Methylation researcher
Data—radiome	OBIA	*https://ngdc.cncb.ac.cn/obia*	A repository for archiving biomedical images and related clinical data	1094	17.01%	General audience
Data—radiome	OPIA	*https://ngdc.cncb.ac.cn/opia*	A platform for archiving and sharing plant image data and i-traits	573 731	1.33%	Plant phenotyping imaging specialist
Data—multimodal	IDP	*https://ngdc.cncb.ac.cn/idp/*	The Immunity Deciphering Project (IDP) big data platform	99 662	–	Immunologist
Data—multimodal	PreDigs	*https://www.biosino.org/predigs/*	A comprehensive resource of context-specific markers for digestive cell annotation	∼3.4 million	–	Oncologist
Data—multimodal	CAVDdb	*https://ngdc.cncb.ac.cn/cavd/*	An integrated multi-omics resource for calcific aortic valve disease (CAVD)	515	–	Cardiologist
Data—multimodal	MTB-KB	*https://ngdc.cncb.ac.cn/mtbkb/*	A literature-curated knowledgebase dedicated to Mycobacterium tuberculosis	74 408	–	Microbiologist
Data—multimodal	ResMicroDb	*https://resmicrodb.cncb.ac.cn/*	A comprehensive database of the respiratory microbiome	106 464	–	Microbiologist
Data—multimodal	TE-SCALE	*https://ngdc.cncb.ac.cn/te-scale/*	A single-cell atlas of transposable element expression across human cancers	330	–	Cancer biologist
Data—multimodal	scMultiModalMap	*https://ngdc.cncb.ac.cn/scmultimodalmap*	A resource for integrating and analyzing single-cell multimodal data	174	–	Omics researcher
Data—multimodal	RDBSB	*https://www.biosino.org/rdbsb*	An open-access resource addressing catalytic biopart data dispersion in synthetic biology	83 193	–	Synthetic biologist
Tool—web server	BIT	*https://ngdc.cncb.ac.cn/bit/*	A cloud-based bioinformatics platform for online computation and analysis	120	41.18%	General audience
Tool—web server	TonguExpert	*https://www.biosino.org/TonguExpert*	A web server for archiving and analyzing tongue images	5992	–	Tongue diagnosis specialist
Tool—web server	miMatch	*https://www.biosino.org/iMAC/mimatch*	A web server designed for microbial metabolic background matching	–	–	Microbiologist
Tool—web server	xMarkerFinder	*https://www.biosino.org/xmarkerfinder*	A web server for identification and validation of cross-cohort biomarkers	–	–	Omics researcher
Tool—web server	VISTA	*https://ngdc.cncb.ac.cn/vista*	A genome-based tool for rapid, scalable virus taxonomy	22 036	21.71%	Virologist
Tool—on-premise tool	Dingent	*https://ngdc.cncb.ac.cn/biocode/tool/BT008001*	A lightweight LLM agent framework for application development	–	–	General audience
Tool—on-premise tool	OmniExtract	*https://ngdc.cncb.ac.cn/biocode/tool/BT007992*	An LLM-based extraction tool for data extraction	–	–	General audience
Tool—on-premise tool	scVar	*https://ngdc.cncb.ac.cn/biocode/tool/BT008000*	A workflow for integrating expression and variation at single-cell resolution	–	–	Single-cell genomics researcher

## Search

### BIG Search

BIG Search (https://ngdc.cncb.ac.cn/search) is a distributed and scalable full-text search engine for a large number of biological resources, providing one-stop cross-database search services for the global research community. In its current version, BIG Search integrates both the NGDC internal databases and 64 partner databases (https://ngdc.cncb.ac.cn/partners), resulting in a total of 1.695 billion data entries and over 1.8 terabytes of data. Furthermore, it supports two mechanisms for automated data index updates: scheduled triggering and change data capture. Additionally, it incorporates 35 NCBI biological databases via e-utilities and 165 EBI biological datasets through API. BIG Search is equipped with advanced search functions and cross-database search services for a wide range of data resources, offering users a more convenient and efficient means for data search and retrieval.

## Knowledge

### Database commons

Database Commons (https://ngdc.cncb.ac.cn/databasecommons) is a curated, categorized catalog of biological databases worldwide, providing impact assessments and valuable statistical insights [40]. Currently, it indexes 7346 biological databases linked to 10 965 publications and 2440 organizations. Based on the average annual citation rate (*z*-index), the top 10 databases are: DAVID [41], KEGG [42], cBioPortal [43], STRING [44], AlphaFold DB [45], UniProt [46], SILVA [47], ENCODE [48], gnomAD [49], and IGSR [50]; the top 10 institutions are: European Bioinformatics Institute, NCBI, National Cancer Institute, Broad Institute, Swiss Institute of Bioinformatics, Kyoto University, Memorial Sloan Kettering Cancer Center, Stanford University, University of Alberta, and Max Planck Institute for Marine Microbiology. Moreover, Database Commons is equipped with a statistical analysis function that can generate quantitative charts upon keyword search, enabling comparisons by country, institution, category, data type, object, and species.

### BioCode

BioCode (https://ngdc.cncb.ac.cn/biocode) is a centralized repository dedicated to archiving bioinformatics tool codes. It features an extensive collection of bioinformatics tools worldwide developed for diverse data analysis purposes, including their names, descriptions, source codes, categories, associated grants, publications, owner information, and organizational affiliations. This year, it has been greatly enhanced by not only allowing any user to submit tools but also retrieving tool information through automated literature curation from leading journals in the bioinformatics field. It has been also further improved by incorporating new features for online tool management and curation. As a result, the current version of BioCode has archived a total of 7520 bioinformatics tools. This centralized archiving has the significant advantage of improving the utility of these bioinformatics tools by making them publicly available, accessible, and usable.

### OpenLB

The Open Library of Bioscience (OpenLB; https://ngdc.cncb.ac.cn/openlb/home) is a scalable and distributed platform designed for storing and retrieving biological literature. In its present version, it incorporates a collection of over 39 million accessible literatures from PubMed (https://pubmed.ncbi.nlm.nih.gov/) [51], bioRxiv (https://www.biorxiv.org/), and medRxiv (https://www.medrxiv.org/). The search algorithm prioritizes key attributes such as publication title and author to enhance retrieval precision and efficiency. The search results page has been optimized to include more comprehensive literature information, such as publication summary and associated data tags, and offers a more user-friendly display of results pagination. Furthermore, it is configured to automatically link with datasets from multiple databases, including GSA, GWH, GenBase, BioProject, BioSample, Database Commons, and BioCode among others, enabling users to easily identify corresponding datasets during literature searches.

## Data

### Raw data and metadata

#### BioProject and BioSample

BioProject (https://ngdc.cncb.ac.cn/bioproject) and BioSample (https://ngdc.cncb.ac.cn/biosample) are two public repositories of biological research projects and samples, respectively. They collect descriptive metadata on biological projects and samples investigated in experiments, enable centralized access to all public projects and samples, and offer cross-links to associated data resources. Up to August 2025, BioProject and BioSample have gathered a total of 32 394 biological projects and 3 046 071 biological samples submitted by 16 797 users, exhibiting a significant growth in contrast to last year’s 20 833 projects and 2 001 551 samples. Regarding international data exchange, these two repositories have mirrored 794 435 projects and 46 031 341 samples of INSDC (International Nucleotide Sequence Database Collaboration) data from NCBI, and shared 15 880 projects and 722 959 samples to INSDC via DNA Data Bank of Japan (DDBJ).

#### GSA and GSA-Human

The Genome Sequence Archive (GSA; https://ngdc.cncb.ac.cn/gsa) [52–54] is an open-access repository designed for the archiving, retrieving, and sharing of non-human raw sequence reads. GSA for Human (GSA-Human; https://ngdc.cncb.ac.cn/gsa-human) [52, 53, 55], a sub-database of GSA, functions as a controlled-access repository, concentrating on human genetic omics data. Till August 2025, GSA and GSA-Human have together accumulated 2 645 702 experiments, 2 889 823 runs, and a total of 83.09 PB of data. Additionally, GSA has integrated 35 805 094 experiments, 37 924 684 runs, and 21.2 PB of raw sequence files from the INSDC’s data resources. GSA maintains close and ongoing engagement with INSDC, actively seeking opportunities to become a member of them and participate in the establishment of international data-sharing policies and data standard specifications. In 2025, INSDC announced its criteria for new membership acceptance. In response, GSA is actively collaborating with DDBJ to develop a data-sharing channel between GSA and DRA, ensuring alignment with these requirements.

#### OMIX

The Open Archive for Miscellaneous Data (OMIX; https://ngdc.cncb.ac.cn/omix) [52, 53], a member of the GSA family, strictly adheres to the FAIR principles and serves as a versatile data repository dedicated to the collection, publication, and sharing of scientific data for biological researchers. As of August 2025, OMIX has archived 8451 datasets comprising 49 432 files, totaling over 154.70 TB of data.

#### GenBase

GenBase (https://ngdc.cncb.ac.cn/genbase) is a user-friendly platform for the archiving, retrieval, and sharing of sequences [56]. As of August 2025, it has received 1 114 470 sequences from 513 submitters, supporting 99 publications, and released 71 499 SARS-CoV-2 genomes. It integrates ∼650 million sequences from INSDC [57] and 390 million sequences from RefSeq [58] for localized data access. GenBase offers batch quality control and annotation tools for influenza virus genomes. In addition, GenBase has developed a sequence validation tool to ensure data accuracy prior to submission and implemented a controlled submission system for human-related sequences. Furthermore, GenBase provides a version comparison function for the sequences and their updates. In the future, GenBase will continue to enhance its data management capabilities and web-based genome annotation tools.

### Genome

#### Genome Warehouse

The Genome Warehouse (GWH; https://ngdc.cncb.ac.cn/gwh) serves as a vital public repository for genome assembly sequences, annotations, and associated metadata [59]. To improve the standardization of genome submissions, GWH has refined its data organization framework for metagenome-assembled genomes (MAGs) and haplotype genomes, and has significantly enriched the metadata associated with MAGs. To minimize redundancy, a feature has been implemented to detect duplicated genomes. Notably, based on reannotation using the Prokaryotic Genome Annotation Pipeline, GWH now integrates AMRFinderPlus [60] to identify genes associated with antimicrobial resistance, stress response, and virulence. GWH supports both Chinese and English interfaces, enhancing accessibility for a broader user base. These updates improve the standardization, interoperability, and reusability of data, thereby adding substantial value to the global genomics research community.

#### Hiland Resource

Hiland Resource (HLR; https://ngdc.cncb.ac.cn/hiland/) is a comprehensive database that integrates phenome, genome, and genetic association data of highland populations worldwide [61]. It systematically integrates data from 29 977 highland individuals across 15 publicly available studies, featuring 29 878 206 genetic variants and 700 342 phenotype-genotype associations spanning 185 traits. HLR offers visualizations of phenotypic patterns across different altitudes, populations, and genders. Additionally, it provides dynamic interfaces for exploring the genetic structure and footprints of natural selection among various highland populations. The genotype-phenotype associations based on highland genome-wide association studies are also integrated and visualized, along with the genome-wide variants and genes. Additionally, HLR provides a user-friendly tool for genotype imputation of high-altitude populations based on a high-quality reference panel of 1001 Tibetan genomes.

#### SugarcaneOmics

SugarcaneOmics (https://ngdc.cncb.ac.cn/scod/) constitutes an indispensable platform addressing critical deficiencies in sugarcane functional genomics and breeding applications, where yield stagnation persists due to the crop’s intricate hybrid polyploid genome [62]. This repository integrates multi-omics datasets spanning 14 sugarcane species and relatives, encompassing genome assemblies, transcriptomic profiles (1256 samples), genetic variations (322 re-sequenced germplasms harbouring ∼175 million variants), and curated functional annotations. Core functionalities incorporate five interactive modules (Genome, Transcriptome, Variome, Feature Genes, and Varieties) alongside specialized toolkits for sequence analysis, gene function elucidation, and CRISPR off-target prediction. The platform facilitates unprecedented cross-species comparative genomics and batch analyses, thereby expediting the translation of genomic discoveries into breeding implementations. This resource holds considerable promise for advancing sustainable agriculture by bridging genomic research and precision breeding for the global scientific and breeding communities.

### Variation

#### GVM

The GVM (https://ngdc.cncb.ac.cn/gvm) [63, 64] (reference in this issue) is a global repository that collects, integrates, and facilitates the submission of genomic variations—including single nucleotide polymorphisms and small insertions/deletions (InDels)—from a wide range of species worldwide. As of August 2025, GVM houses ∼2.09 billion variants from 73 species, deriving from 437 projects and 101 967 samples, all of which have been manually curated and processed through a standardized analysis pipeline. Meanwhile, it has archived 880 data submissions covering 754 879 samples across 78 species submitted from 182 organizations. In this version, a new model with deleterious variant information and population genetic selection signals has been launched. A new online tool (VersionMap) is introduced, which enables convenient mapping of variants across different genome assemblies. Collectively, these updates will strengthen GVM’s capacity and enhance its value as a global genomic variation resource.

#### TOAnnoPriDB

TOAnnoPriDB (http://bigdata.ibp.ac.cn/TOAnnoPriDB) is a comprehensive resource for annotation and prioritization of non-coding variants across human genome [65]. Based on the NyuWa genome resource [66], TOAnnoPriDB aims to help emphasize the potential functional effects of variants and the relationship between variants and human disease. TOAnnoPriDB covers ∼98% of the non-coding region of human genome in total and integrates trans-omic information from 147 public resources, including several databases we developed previously, namely NCVD [66–70], NONCODE [71], NPInter [72], piRBase [73], SmProt [74], and LncVar [75]. Based on the data integration, TOAnnoPriDB constructs a framework to prioritize variations according to evidence that supports the functional impact of variants and provides a user-friendly web interface to help users search and analyze variants. JBrowse 2 is incorporated to visualize annotation information. The allele frequency, gene expression, and molecular interaction related to the variations are also visualized. TOAnnoPriDB can serve as a powerful tool for variant annotation and prioritization, which can help users explore and understand the association between non-coding variants and human diseases.

### Transcriptome

#### Gene Expression Nebulas

Gene Expression Nebulas (GEN; https://ngdc.cncb.ac.cn/gen) is a data portal integrating transcriptomic profiles from both bulk and single-cell levels in various conditions across multiple species [76]. As of August 2025, GEN has collectively accumulated 91 species, 629 datasets, 86 584 samples, 19 281 005 cells, and 584 publications, marking a significant increase compared to the previous version. This year, we have systematically incorporated 50 new species to expand the species diversity, including 40 plants, 5 animals, and 5 protists. Moreover, 26 816 samples related to aplastic anemia and Alzheimer’s disease have been integrated and analyzed to enrich the data volume. Additionally, we have updated the GENToolkit to encompass standardized analyses of global gene expression profiles, differential gene expression, and functional enrichment, offering more comprehensive and multi-tiered analytical and visualization capabilities, along with improved robustness and user-friendliness.

#### LncExpDB

LncExpDB (https://ngdc.cncb.ac.cn/lncexpdb) is a comprehensive resource that integrates and rigorously curates human long non-coding RNA (lncRNA) expression profiles across diverse biological contexts [77] (reference in this issue). Built upon the standardized gene reference from LncBook [78], it evaluates expression reliability, highlights featured genes, and identifies lncRNA–messenger RNA (mRNA) interactions by co-expression networks. This year’s update introduces three additional biological contexts: neurodegenerative disease, reproduction, and wound healing. It also incorporates two newly developed tools: LncNet for visualizing and exploring lncRNA–mRNA co-expression networks and LncImm for examining lncRNA correlations with immune checkpoint genes across four cancers with immunotherapy data. These enhancements are part of the major release of LncExpDB 2.0, launched in 2025. Compared with version 1.0, launched in 2020, LncExpDB 2.0 expands the data coverage from 9 to 15 biological contexts, identifying 39 253 featured genes (among 101 293) and >28 million co-expression pairs. It further enhances functionality with new analysis tools, a pipeline module, and in-depth functional analyses.

#### CROST

CROST (https://ngdc.cncb.ac.cn/crost) integrates data, knowledge, and tools for spatial transcriptomics [79]. Since August 2023, it has added three model organisms (*Sus scrofa, Gallus gallus, Oryctolagus cuniculus*) and expanded datasets covering disease, environmental, and developmental contexts. A new tool, SCAN, was introduced for cell type annotation. CROST now contains 1313 high-quality samples from 234 projects and 11 species, a 30% increase over the previous version. SCAN integrates six marker databases to provide predictions comparable to manual annotation, and users can upload data and export results. Together with SpatialAP, CROST enables one-stop single-cell and spatial transcriptomic analysis, offering a comprehensive view of tissue architecture and advancing understanding of biological mechanisms, particularly in tumors.

#### TWAS Atlas

TWAS Atlas (https://ngdc.cncb.ac.cn/twas/) is a comprehensive data resource that systematically consolidates published transcriptome-wide association study (TWAS) findings [80]. Recently, we curated high-quality gene-trait associations and expanded phenotypic coverage by conducting TWAS analysis based on public GWAS datasets. Overall, the number of associations increased by 274 972, derived from 224 publications and 171 datasets. Regarding feature updates, we improved the interactive visualization in TWAS Atlas 2.0. The Knowledge Graph now includes supporting evidence descriptions extracted from original publications. Furthermore, we added multidimensional analysis modules, including functional enrichment, Mendelian randomization, colocalization, and fine-mapping, providing researchers with a comprehensive toolkit to investigate causal gene-trait relationships. TWAS Atlas 2.0 offers a comprehensive platform for identifying risk loci for complex traits and exploring potential regulatory mechanisms underlying various diseases.

#### TEDD

TEDD (https://ngdc.cncb.ac.cn/tedd) is a publicly accessible database dedicated to the systematic collection, analysis, and visualization of human translation efficiency dynamics (TE, TR, EVI) with a strong emphasis on regulatory 5′/3′ UTR features (reference in this issue). The current release contains 279 datasets comprising 1518 samples from 143 projects, spanning 24 tissues/cell types, 74 cell lines, and 52 conditions. The translation efficiency dynamics metrics are calculated at both the gene and transcript levels, complemented by detailed UTR annotations to support fine-grained analysis of translational regulation. An integrated online analysis platform allows multidimensional comparisons of TE/TR/EVI across genes, transcripts, KEGG/GO-defined gene sets, and UTR elements. Overall, TEDD provides a comprehensive resource for advancing both basic research and translational applications in translatomics—particularly in areas such as mRNA vaccine development, synthetic biology, gene therapy, and enzyme engineering—by supporting rational design of gene expression systems for efficient protein production.

### Epigenome

#### EWAS Open Platform

EWAS Open Platform (https://ngdc.cncb.ac.cn/ewas) serves as a continuously updated resource dedicated to epigenome-wide association studies (EWAS), integrating data, knowledge, and toolkit [81] (reference in this issue). A unified retrieval function and an AI-based Q&A Assistant have been introduced, substantially enhancing the efficiency of cross-module knowledge retrieval and interactive exploration. On the data side, GMQN, a batch effect correction tool optimized for the latest DNA methylation 935K arrays, has been upgraded [82] and 20 373 new high-quality samples have been incorporated into EWAS Data Hub [83]. On the knowledge side, EWAS Open Platform has added 54 667 epigenetic associations curated from publications [84], and an interactive multi-omics regulatory network integrating 17 494 causal relationships among DNA methylation, gene expression, and diverse traits. These updates aim to facilitate deeper exploration of the regulatory mechanisms underlying disease onset and progression.

#### MethBank

The Methylation Bank (MethBank; https://ngdc.cncb.ac.cn/methbank/) [85–87] is a comprehensive repository of whole-genome, single-base resolution DNA methylation across multiple species and biological contexts. To support metadata standardization, AI-assisted Metadata Curation System that integrates pretrained LLMs with ontology hierarchies has been implemented, enabling precise alignment of attributes such as tissue/cell line and disease. Plant-specific attributes, including geographic location, isolate, ecotype, and generation, have also been incorporated. In its latest release, MethBank documents a 49.08% increase in data volume, with the addition of 588 human samples (*Homo sapiens*) and 1156 plant samples (*Arabidopsis thaliana*). As of August 2025, MethBank integrates ~648 million gene-level methylation profiles from 26 species, which are derived from 410 high-quality projects and 5297 samples, and curated under a structured metadata framework with standardized analytical pipelines to ensure reproducibility and cross-study comparability.

### Radiome

#### OBIA

The Open Biomedical Imaging Archive (OBIA; https://ngdc.cncb.ac.cn/obia) [52, 88] is a repository of biomedical images alongside its clinical data. Data are organized into five hierarchical objects—Collection, Individual, Study, Series, and Image—with Individuals linked to GSA-Human via accession numbers to enable multi-omics research. To ensure data privacy and integrity, OBIA implements standardized de-identification and quality control procedures and provides both open and controlled access. Beyond conventional web-based querying and browsing, OBIA supports advanced image retrieval. As of September 2025, OBIA has housed a total of 1094 individuals, 4349 studies, 24 918 series, and 2 012 007 images covering 9 modalities and 30 anatomical sites.

#### OPIA

The OPIA (https://ngdc.cncb.ac.cn/opia/) [89] is an open-access platform for archiving and sharing plant image datasets and associated image-based traits (i-traits) derived from high-throughput phenotyping technologies. As of August 2025, OPIA hosts 89 datasets from 43 plant species, comprising 573 731 images and 2 424 692 labeled instances. These datasets are AI-ready, supporting classification and identification tasks. OPIA also offers a suite of online tools for image pre-processing and intelligent phenotypic analysis. Among them, Img2Variety is a new CNN-based framework for crop accession identification using whole-plant images across all developmental stages. It achieves an accuracy of 88.66% for rice and ∼80% for maize intraspecific variety identification. In summary, OPIA is a useful resource for advancing crop research, enhancing breeding efficiency, and ultimately driving agricultural progress.

### Multimodal

#### IDP

Immunity Deciphering Project (IDP; https://ngdc.cncb.ac.cn/idp/) is a comprehensive resource for the submission, management, integration, and sharing of immunity-related data, designed to facilitate the systematic digital decoding of human immunity. Supported by the National Natural Science Foundation of China’s major research program, the platform has established standardized terminology and data management systems and has facilitated the submission of 42 projects, encompassing 11 246 samples with 42.68 TB of data. Building on this foundation, IDP has standardized and integrated 617 multi-source and multi-modal datasets, covering 92 662 samples across 61 disease types. These datasets span diverse modalities, including scRNA-seq, scBCR-seq, scTCR-seq, ATAC-seq, Bisulfite-seq, and Hi-C, and are made accessible through cross-type search and metadata download functionalities. Overall, IDP provides high-quality data submission and sharing support for understanding the immune system and enabling immune interventions and personalized medicine.

#### PreDigs

PreDigs (https://www.biosino.org/predigs/) is a user-friendly database of predicted cell-type signatures in the digestive system, integrating single-cell RNA sequencing data from gastrointestinal tumors to explore cellular composition and heterogeneity [90]. It contains 124 curated datasets encompassing over 3.4 million cells, all of which are available for download. Subtype labels are unified, and a cell ontology tree was constructed with 142 cell types across eight hierarchical levels. Meanwhile, we calculated three different context-specific cell-type markers, including “Cell Markers,” “Subtype Markers,” and “TPN Markers,” based on various application requirements within or across tissues. Through the integrated analysis of PreDigs data, we identified distinct cell subpopulations exclusive to tumors, one of which corresponds to tumor-specific endothelial cells. Additionally, PreDigs offers online cell annotation tools, allowing users to classify single cells with greater flexibility. Overall, the database provides valuable insights into tumor heterogeneity and its impact on disease progression and treatment response.

#### CAVDdb

Calcific Aortic Valve Disease Database (CAVDdb; https://ngdc.cncb.ac.cn/cavd/) is an integrated multi-omics resource for calcific aortic valve disease (CAVD), the leading cause of aortic stenosis worldwide. The current release consolidates diverse datasets from human tissues and cell line models, including 24 projects and 515 samples. Specifically, it integrates transcriptomic data from 14 BioProjects and 214 BioSamples, covering 35 894 genes in tissues and 19 297 genes in cell lines, with 5453 differentially expressed genes across five groups. It also includes proteomic profiles of 5234 proteins (303 differentially expressed), metabolomic data of 480 metabolites (214 differentially expressed), single-cell transcriptomic data from 145 045 cells across six cell types, and epigenomic modules including DNA methylation levels of 78 311 genes from aortic valve samples, as well as one ATAC-seq and six ChIP-seq samples. CAVDdb offers three interactive tools, including a genome browser and functional enrichment modules. Overall, CAVDdb provides a comprehensive resource, facilitating mechanism studies and therapeutic target discovery.

#### MTB-KB

MTB-KB (https://ngdc.cncb.ac.cn/mtbkb/) is the first literature-curated knowledgebase dedicated to *Mycobacterium tuberculosis* (MTB) and tuberculosis (TB). It integrates 74 408 high-confidence associations manually extracted from 1187 publications, including 18 232 biological entities across eight major sections: Epidemiology, Diagnosis, Drug, Regimen, Vaccine, Drug Resistance, Virulence Factor, and Immune Mechanism. All entities are annotated and standardized using authoritative resources, thereby improving consistency, interoperability, and clinical relevance. Especially, it constructs an interactive knowledge graph that reveals cross-sectional relationships regarding MTB-host interactions, treatment strategies, and vaccine development opportunities, enabling multi-dimensional analysis, association, and inference. MTB-KB also features user-friendly modules for section-based browsing, quick and advanced search, statistical visualization, and data download. MTB-KB fills a critical gap in TB research by systematically consolidating literature-based knowledge and enhancing its interpretability through standardized annotation and graph analytics, providing a valuable and innovative platform that supports basic research, translational applications, and global TB control efforts.

#### ResMicroDb

ResMicroDb (https://resmicrodb.cncb.ac.cn/) is a comprehensive database and analysis platform dedicated to human respiratory microbiome research. It integrates 106 464 samples from 514 projects, spanning 10 respiratory tract sites and 146 phenotypes, along with 31 curated metadata fields (reference in this issue). The database also includes 11 908 microbe-disease associations identified from 132 case-control studies. ResMicroDb features a user-friendly web interface for querying, browsing, visualizing, and analyzing respiratory microbiome data. It provides three integrated analytical tools: Microbiome Composition, for visualizing taxonomic profiles; Sample Similarity Search, for inferring sample characteristics through similarity-based comparisons; and Cross-study Analysis, for identifying shared and specific microbial features across cohorts, sites, and diseases. In summary, ResMicroDb serves as a vital resource for advancing research on the respiratory microbiome and its clinical applications.

#### TE-SCALE

TE-SCALE (https://ngdc.cncb.ac.cn/te-scale/) is a comprehensive database for exploring transposable element (TE) expression across human cancers at single-cell resolution, addressing the underrepresentation of TEs in conventional gene-centric analyses (reference in this issue). It is constructed from publicly available single-cell transcriptomic datasets comprising over 1.3 million high-quality cells from 330 samples across 20 cancer types and 12 tissue origins. Powered by the in-house streamlined computational pipeline *scTEfinder*, TE-SCALE enables robust quantification of 1051 curated TE subfamilies, integrates gene and TE expression profiles, and provides precise cell-type annotation. It delivers a pan-cancer TE expression atlas with interactive, multi-scale exploration and three analytical modules: differential TE expression, TE-gene co-expression network, and functional enrichment. Notably, TE-SCALE is the first database, to our knowledge, that identifies tumor-specific TEs preferentially expressed in particular cancer types or pathological states, underscoring their potential as biomarkers for diagnosis, disease monitoring, and immunotherapeutic targeting.

#### scMultiModalMap

The single-cell multimodal data map (scMultiModalMap; https://ngdc.cncb.ac.cn/scmultimodalmap/) is a comprehensive resource for collecting, integrating, visualizing, and analyzing single-cell multimodal data. It currently includes 174 datasets covering two types of multimodality, 15 sequencing protocols, 3 antibody panels, and 79 conditions, encompassing over 3.2 million cells. Users can access detailed information about each dataset, including sample quality control, multimodal integration-based UMAPs, differential analyses (gene expression, protein abundance, and chromatin accessibility), and enrichment analysis results. Seven online analytical modules are available, consisting of two single-modality modules, three cross-modality modules, and two cell-level modules. These modules enable users to visualize modality-specific features across different cell types, explore relationships among features across various modalities, and analyze changes in cell composition or cell–cell communication events related to biological environments. Overall, scMultiModalMap serves as a crucial resource for exploring single-cell multimodal data, enabling users to understand cellular heterogeneity and advance their knowledge of cellular functions.

#### RDBSB

The Registry and Database of Bioparts for Synthetic Biology (RDBSB; https://www.biosino.org/rdbsb/) is a comprehensive, open-access resource addressing the dispersion of catalytic biopart functional data across databases and literature, a key barrier to designing metabolic pathways and optimizing cell factories in synthetic biology [91]. In its current release, RDBSB systematically curates 83 193 catalytic bioparts with experimental evidence, providing detailed qualitative and quantitative catalytic information, such as activities, substrates, optimal pH/temperature, and chassis specificity. The platform features an interactive search engine, visualization tools, and practical utilities like biopart finder, structure prediction, and pathway design tools, directly supporting synthetic biology workflow needs. Additionally, RDBSB supports community-driven biopart submission, which has facilitated over 1000 user contributions to date, enabling rapid data sharing. As a freely available resource, RDBSB significantly enriches pathway design resources and serves as an essential tool for advancing synthetic biology research, from basic biopart characterization to applied cell factory optimization.

## Tool

### Web server

#### BIT

Bioinformatics Toolkits (BIT; https://ngdc.cncb.ac.cn/bit/) is a cloud-based bioinformatics platform for online computing and analysis. It integrates a variety of frequently used bioinformatics tools, allowing users to select the tools they need, upload data, customize parameters, and quickly complete various analysis tasks. Users can also browse and download the analysis results. The current version of the software integrates 10 categories of tools, including visualization, sequence alignment, image processing, and omics data analysis, among others, and deploys 120 analysis tools in total. BIT offers a user manual that provides operational guidance and usage examples to facilitate quick initiation. It also features a tool navigation bar and search functionality to help users quickly locate the tools they need, thereby enhancing the user experience.

#### TonguExpert

TonguExpert (https://www.biosino.org/TonguExpert) is a free automated platform for archiving, analyzing, and extracting detailed phenotypes from tongue images to enhance the objectivity and accuracy of tongue diagnosis in clinical and research settings [92]. It integrates deep learning algorithms (such as YOLOv8 and ResNet50) with refined preprocessing, hosting the largest publicly available tongue image database to date, comprising 5992 high-quality images from a Chinese population, and has established a fine-grained phenotype library containing 773 phenotypes—355 global phenotypes from the whole tongue, tongue body, and tongue coating, and 408 local phenotypes from tongue fissures and tooth marks. TonguExpert demonstrates high predictive performance for four Traditional Chinese Medicine (TCM) phenotypes (ROC-AUC 0.89–0.99 for color) and exhibits strong generalization to novel phenotypes such as greasy coating. TonguExpert provides a user-friendly web interface for image upload and phenotype extraction, advancing automated, interpretable tongue diagnosis for TCM and precision health research.

#### miMatch

miMatch (https://www.biosino.org/iMAC/mimatch) is a web server designed to promote the integration of metagenomic cohorts and strengthen causal relationships in metagenomic research [93]. Low concordance across metagenomic studies remains a major challenge, largely due to host-related confounders such as genetics, environment, and lifestyle. To address this, miMatch leverages the microbial metabolic background as a comprehensive reference for host-related variables and applies propensity score matching to construct balanced case-control pairs. miMatch shows robust performance in both simulated and real-world data, effectively reducing false positives in microbial signature detection and enhancing result consistency and model generalizability across cohorts. The web server provides an easy-to-use platform for applying this framework, enabling users to easily incorporate miMatch into their studies. By constructing well-matched cohorts, miMatch helps researchers generate more reliable and generalizable insights into microbiome-disease associations.

#### xMarkerFinder

xMarkerFinder (https://www.biosino.org/xmarkerfinder/) is a computational platform for robust biomarker discovery across heterogeneous multi-cohort datasets [94]. Variability introduced by different cohorts, sequencing platforms, and study designs often limits reproducibility in microbiome and other omics research. xMarkerFinder addresses this challenge by integrating statistical modeling, cohort harmonization, and rigorous feature selection to minimize batch effects and ensure cross-study consistency. Primarily designed for microbiome analysis, it can also be extended to other omics, phenotypic, and environmental datasets. The web server provides both a streamlined one-click workflow for rapid biomarker identification and a customizable stepwise mode for interactive exploration. Curated metadata from diverse publicly available microbiome studies allows researchers to efficiently identify and select relevant datasets and facilitates data reuse. By combining reproducibility, scalability, and accessibility, xMarkerFinder offers a versatile platform for reliable biomarker discovery and translational applications.

#### VISTA

VISTA (https://ngdc.cncb.ac.cn/vista) is a computational tool that integrates a novel pairwise sequence-comparison system with an automatic threshold-identification framework for virus taxonomy [95]. By leveraging k-mer profiles, physico-chemical property sequences, and machine-learning techniques, VISTA constructs a robust distance-based model for taxonomic assignment. The tool has been applied to 22 036 complete viral genomes, covering six Baltimore classes, the class *Caudoviricetes*, and 39 additional families, demonstrating scalability across both prokaryotic and eukaryotic viruses. Importantly, VISTA also assigns previously unclassified viral genomes, providing objective species- or genus-level assignment. Application to 679 unclassified prokaryotic genomes from metagenomic datasets led to the recognition of 46 novel virus families [95]. VISTA-generated demarcation thresholds constitute data-driven “gold standards” for genus and species boundaries and are now being promoted for families such as *Arteriviridae, Dicistroviridae*, and *Filoviridae*. Overall, VISTA provides an efficient framework for genome-based virus classification and facilitates the integration of newly sequenced genomes into official taxonomy frameworks.

### On-premise

#### Dingent

Dingent (https://ngdc.cncb.ac.cn/biocode/tool/BT008001) is a lightweight, user-friendly agent framework designed to simplify and accelerate the development of agent applications powered by LLMs. It integrated a backend service (LangGraph; https://www.langchain.com/langgraph), a ready-to-use chat interface, and a full-featured admin dashboard, significantly reducing the amount of repetitive “glue code” typically needed when building agent-based applications. A key feature of Dingent is its powerful web-based admin dashboard, which enables users to configure assistants, build workflows, and adjust settings through an intuitive graphical interface, eliminating the need for manual file editing. Additionally, Dingent supports instant project initialization with a single command and offers an extensible plugin system for incorporating custom tools. Overall, by streamlines the development process, Dingent allows developers to concentrate on core logic and facilitates the efficient creation of sophisticated, customizable AI agents.

#### OmniExtract

OmniExtract (https://ngdc.cncb.ac.cn/biocode/tool/BT007992) is an LLM-based automatic extraction tool specifically designed for information extraction from literature and documents. It utilizes prompt optimization engineering to enhance extraction performance based on curated data and provides various file format parsing tools. The tool supports batch extraction of multi-property entities from original documents (such as PDF or XML) as well as tabular files. OmniExtract offers a user-friendly approach: the entire extraction process, including model configuration, file parsing, prompt optimization, and information extraction, can be easily customized by modifying configuration files. Evaluation results show that when using open-source models, OmniExtract achieves a high accuracy with ranges from 82.63% to 89.00% across three public datasets: the WikiReading Recycled dataset [96], the CriticalCoolingRates dataset [97], and the Yidu-S4K dataset. Moreover, when applied to extract phenotypic information from dog breed standard documents, the accuracy exceeds 90% after optimization. Overall, OmniExtract provides convenient and reliable information extraction functionality with consistently stable performance.

#### scVar

scVar (https://ngdc.cncb.ac.cn/biocode/tool/BT008000) is a comprehensive workflow for the detection and functional characterization of single-nucleotide variants from 10× Genomic scRNA-seq data. This tool demonstrates high accuracy in identifying somatic mutations, exhibiting enhanced sensitivity particularly for low-frequency variants, and operates effectively without requiring paired normal samples or predefined cell-type annotations. Subsequent to variant detection, scVar integrates multiple databases to perform functional and clinical annotations of the identified variants. Additionally, the workflow encompasses a suite of downstream analytical modules tailored to distinct cellular subpopulations, including mutational signature analysis, quantification of tumor mutational burden and clonal diversity, functional enrichment analysis of mutated genes, and differential gene expression profiling within mutated cells. Overall, scVar improves the accuracy of identifying low-frequency variants and enables joint analysis of expression and variation at single-cell resolution, offering new insights into tumor heterogeneity.

## Concluding remarks

Amid the exponential growth of multi-omics and multi-modal data, CNCB–NGDC is committed to providing an increasingly comprehensive and intelligent suite of database resources, facilitating efficient, high-quality data archiving, integration, and utilization, and offering broad support to the global research community to drive transformative advances in life, health, and medical sciences. Looking ahead, CNCB–NGDC will continue to deeply integrate AI technologies to enhance the management, integration, and analysis of multi-omics data, optimize big data storage and computing platforms, and develop efficient analytical tools and pipelines for addressing complex biological questions. These efforts are expected to accelerate knowledge discoveries in genomics, molecular biology, and biotechnology, foster cross-disciplinary research and innovation, and support a wide range of applications worldwide in personalized medicine, precision diagnostics, drug discovery, crop breeding, and biosafety.

## Data Availability

All resources and services are publicly available on the home page of CNCB–NGDC (https://ngdc.cncb.ac.cn).

## Appendix

**Corresponding author**: Yiming Bao^1,2,3,*^

**Co-corresponding authors:** Zhang Zhang^1,2,3,*^, Wenming Zhao^1,2,3,*^, Jingfa Xiao^1,2,3,*^, Shuhui Song^1,2,3,*^, Shunmin He^3,4,*^, Guoqing Zhang^5,6,*^, Yixue Li^5,7,*^, Guoping Zhao^5,8,*^, Runsheng Chen^3,4,*^

**CNCB–NGDC MEMBERS** (Arranged by project role and then by contribution except for Team Leader (TL), as indicated)

**Hiland Resource:** Yibo Wang^1,2,3,#^, Weijie Zhang^3,9,#^, Xiaoning Chen^1,2,3,#^, Yanling Sun^1,2,#^, Bixia Tang^1,2^, Yu Zhang^9^, Kai Liu^3,9^, Wenming Zhao^1,2,3,*^ (TL), Bing Su^9,10^ (TL), Yaoxi He^9,10^ (TL)

**TOAnnoPriDB:** Tingrui Song^4,#^, Yirong Shi^3,4,#^, Yanyan Li^4^, Di Hao^4^, Kaixin Zhan^4^, Tao Xu^11,12^, Runsheng Chen^3,4,*^, Shunmin He^3,4,*^

**TEDD:** Wenyan Lei^2,3,13,#^, Cuidan Li^2,13,#^, Hengyu Zhou^2,13,14,#^, Zhi Nie^1,2,3,#^, Anke Wang^1,2,#^, Pan Li^2,3,13^, Peihan Wang^2,3,13^, Zhuojing Fan^1,2^, Rongxi Zhu^15^, Haoyu Cheng^16^, Yuxian Guo^15^, Liya Yue^2,13^, Xiaoyuan Jiang^2,13^, Renjun Gao^16^, Yongjie Sheng^16^, Haitao Niu^17^, Tuohetaerbaike·Bahetibieke^18^, Wenbao Zhang^18^, Wenming Zhao^1,2,3^, Jingfa Xiao^1,2,3,*^ (TL), Fei Chen^2,3,13,17,18,#^ (TL)

**PreDigs:** Jiayue Meng^5,#^, Mengyao Han^19,#^, Yuwei Huang^5,#^, Liyun Yuan^5,#^, Guoqing Zhang^5,6,*^

**scMultiModalMap:** Zhi Nie^1,2,3,#^, RuiKun Xue^1,2,3,#^, Zhuojing Fan^1,2^, Jingfa Xiao^1,2,3,*^, Jingyao Zeng^1,2,#^

**TE-SCALE:** Xini Meng^2,3,13,#^, Zhi Nie^1,2,3,#^, Qifei Wang^2,3,13^, Yiwen Hu^2,3,13^, Yulan Deng^20,21^, Na Ai^2,13,22,23^, Zheng Huang^2,3,13^, Yun Li^2,3,13^, Yang Yuan^3,24^, Jingfa Xiao^1,2,3^, Jingyao Zeng^1,2,#^, Guochao Li^2,3,13,#^, Lan Jiang^2,3,13,25,#^

**TonguExpert:** Ting Li^5,#^, Ling Zuo^5,26,#^, Jianxin Chen^26,27,#^, Qianqian Peng^5,#^, Guoqing Zhang^5,6,*^, Sijia Wang^5,28,#^

**CAVDdb:** Sicheng Wu^1,2,3,#^, Hao Jiang^29,30,#^, Yibo Wang^1,2,3,#^, Hailong Kang^1,2,3^, Jiawei Shi^31^, Bixia Tang^1,2^, Nianguo Dong^31^ (TL), Wenming Zhao^1,2,3,*^ (TL), Ximiao He^29,30,32^ (TL)

**IDP:** Fei Yang^1,2,#^, Shuai Jiang^33,#^, Zhenxian Han^1,2,#^, Xue Bai^1,2,#^, Dong Zou^1,2^, Sisi Zhang^1,2^, Yi Wang^1,2,3^, Zhijian Duan^1,2,3^, Lun Li^1,2^, Zhuojing Fan^1,2^, Shuhui Song^1,2,3,*^

**MTB-KB:** Pan Li^2,3,13,#^, Cuidan Li^2,13,#^ (TL), Rongxi Zhu^15^, Wenjing Sun^16^, Hengyu Zhou^2,13,14^, Zhuojing Fan^1,2^, Liya Yue^2,13^, Sijia Zhang^16^, Xiaoyuan Jiang^2,13^, Quan Luo^16^, Jinying Han^16^, Hairong Huang^34^, Adong Shen^35^, Tuohetaerbaike·Bahetibieke^18^, Jing Wang^18^, Wenbao Zhang^18^, Hao Wen^18^, Haitao Niu^17^, Congfan Bu^1,2^, Zhang Zhang^1,2,3^, Jingfa Xiao^1,2,3^, Renjun Gao^16,#^ (TL), Fei Chen^2,3,13,17,18,#^ (TL)

**ResMicroDb:** Xiaotong Ji^1,2,3,36,#^, Qiheng Qian^1,2,#^, Hao Zhang^1,2,3,#^, Qingyun Cai^1,2,3,#^, Kaiwen Zhang^1,2,3^, Jingfa Xiao^1,2,3^, Xiaoqing Jiang^1,2,#^, Mingkun Li^2,3,13,#^

**SugarcaneOmics:** Hong Luo^1,2,#^, Xue Bai^1,2,#^, Zishan Wu^1,2,3,#^, Siwei Ren^1,2,3^, Haixia Xie^1,2,3^, Zhixiang Yuan^1,2^, Dongmei Tian^1,2,#^, Shuhui Song^1,2,3,*^

**Dingent:** Demian Kong^1,2,3,#^, Shaoqi Bei^1,2,3^, Yueyue Wu^1,2,3^, Bixia Tang^1,2,#^ (TL), Wenming Zhao^1,2,3,*^ (TL)

**miMatch:** Lei Liu^37,#^, Suqi Cao^38,#^, Weili Lin^37,#^, Guoqing Zhang^5,6,*^, Ruixin Zhu^37,#^, Dingfeng Wu^38,#^

**OmniExtract:** Yibo Wang^1,2,3,#^, Bixia Tang^1,2,#^ (TL), Sicheng Wu^1,2,3^, Yuyan Meng^1,2,3^, Demian Kong^1,2,3^, Wenming Zhao^1,2,3,*^ (TL)

**RDBSB:** Wan Liu^5,#^, Pingping Wang^39,#^, Xinhao Zhuang^5,#^, Xing Yan^40,#^, Zhihua Zhou^39,#^, Guoqing Zhang^5,6,*^

**xMarkerFinder:** Wenxing Gao^37,#^, Weili Lin^37,#^, Qiang Li^5,#^, Guoqing Zhang^5,6,*^, Ruixin Zhu^37,#^, Na Jiao^38,41,#^

**VISTA:** Yiyun Liu^1,2,3,#^, Lili Tian^1,2,3,#^, Yiming Bao^1,2,3,*^

**scVar:** Wei Zhao^1,2,3,#^, Fei Yang^1,2^, Wenting Zong^1,2^, Xinchang Zheng^1,2,#^, Yiming Bao^1,2,3,*^

**BioProject & BioSample & GSA & GSA-Human:** Xu Chen^1,2,#^, Tingting Chen^1,2,#^, Sisi Zhang^1,2,#^, Xiaolong Zhang^1,2,#^, Yubo Zhou^1,2^, Anke Wang^1,2^, Junwei Zhu^1,2^, Bing Xu^1,2^, Lili Dong^1,2^, Caixia Yu^1,2^, Wenjie Li^1,2^, Zhuojing Fan^1,2^, Shuang Zhai^1,2^, Yubin Sun^1,2^, Qiancheng Chen^1,2^, Yanqing Wang^1,2,#^ (TL), Wenming Zhao^1,2,3,*^ (TL)

**OMIX:** Anke Wang^1,2,#^, Caixia Yu^1,2,#^, Yanqing Wang^1,2^, Sisi Zhang^1,2,#^ (TL)

**GenBase:** Congfan Bu^1,2,#^, Xuetong Zhao^1,2,#^, Xue Bai^1,2,#^, Jingfa Xiao^1,2,3^, Zhang Zhang^1,2,3^, Wenming Zhao^1,2,3^, Bixia Tang^1,2,#^ (TL), Yiming Bao^1,2,3,*^

**Database Commons:** Shaosen Zhang^1,2,3,#^, Dong Zou^1,2,#^, Jinbiao Wang^1,2,3,#^, Yuhao Zeng^1,2,3,#^, Zheng Luo^1,2,3,#^, Yiran Zhan^1,2,3^, Zihan Wang^1,2,3,36^, Xi Zhao^1,2,3^, Yuxi Liu^1,2,3^, Lina Ma^1,2,3,#^ (TL)

**Genome Warehouse:** Xuetong Zhao^1,2,#^, Zhenxian Han^1,2,#^, Yingke Ma^1,2,#^, Meili Chen^1,2,3,#^ (TL)

**GVM:** Dongmei Tian^1,2,#^, Xue Bai^1,2,#^, Haixia Xie^1,2,3,#^, Siwei Ren^1,2,3,#^, Bixia Tang^1,2,#^, Shuhui Song^1,2,3,*^ (TL)

**Gene Expression Nebulas:** Tongtong Zhu^1,2,#^, Wenzhuo Cheng^1,2,3,#^, Yuan Chu^1,2,3,#^, Dong Zou^1,2,#^, Zhenxian Han^1,2,#^, Ming Chen^1,2,3^, Zihan Wang^1,2,3,36^, Yiran Zhan^1,2,3^, Zheng Luo^1,2,3^, Tianyi Xu^1,2,#^(TL), Zhang Zhang^1,2,3,*^

**TWAS Atlas:** Hao Gao^1,2,3,#^, Si Zheng^42,43,#^, Congfan Bu^1,2,#^, Jialin Mai^1,2,3,#^, Jinbei Wang^42^, Rui Tang^42^, Jingyao Zeng^1,2,#^, Jiao Li^42,#^, Jingfa Xiao^1,2,3,*^

**LncExpDB:** Yue Qi^1,2,3,#^, Zhao Li^1,2,3^, Lina Ma^1,2,3,#^ (TL)

**EWAS Open Platform:** Fei Yang^1,2,#^, Wenting Zong^1,2,#^, Zhuang Xiong^44,#^, Demian Kong^1,2,3,#^, Bixia Tang^1,2^, Xupeng Chen^44^, Yaoke Wei^1,2,3^, Xiangyu Yu^1,2,3^, Yiran Zhang^1,2,3^, Rujiao Li^1,2,3,#^ (TL)

**MethBank:** Mochen Zhang^1,2,#^, Yaoke Wei^1,2,3,#^, Huiying Chen^1,2,3,36,#^, Rujiao Li^1,2,3,#^ (TL)

**CROST:** Guoliang Wang^2,3,13,#^, Song Wu^1,2,3,#^, Hongzhu Qu^2,3,13,#^ (TL), Yiming Bao^1,2,3,*^ (TL), Xiangdong Fang^2,3,13,#^ (TL)

**OBIA:** Enhui Jin^1,2,3,#^, Dongli Zhao^45,#^, Gangao Wu^1,2,3,#^, Junwei Zhu^1,2^, Zhonghuang Wang^1,2,3^, Zhiyao Wei^45^, Sisi Zhang^1,2^, Anke Wang^1,2^, Bixia Tang^1,2^, Xu Chen^1,2^, Yanling Sun^1,2,#^, Zhe Zhang^46,#^, Wenming Zhao^1,2,3,*^, Yuanguang Meng^45,46,#^

**OPIA:** Yongrong Cao^1,2,3,#^, Dongmei Tian^1,2,#^, Shuhui Song^1,2,3,*^

**BIG Search:** Dong Zou^1,2,#^ (TL), Zhixiang Yuan^1,2^, Zhang Zhang^1,2,3,*^

**BioCode:** Dong Zou^1,2,#^ (TL), Zhixiang Yuan^1,2,#^, Tianyi Xu^1,2^, Zhang Zhang^1,2,3,*^

**BIT:** Dong Zou^1,2,#^ (TL), Zhenxian Han^1,2,#^, Zhixiang Yuan^1,2^, Zhang Zhang^1,2,3,*^

**OpenLB:** Dong Zou^1,2,#^ (TL), Zhang Zhang^1,2,3,*^

**Writing Group:** Mochen Zhang^1,2,#^, Fei Yang^1,2,#^, Zhuojing Fan^1,2^, Shuhui Song^1,2,3,*^, Wenming Zhao^1,2,3,*^, Jingfa Xiao^1,2,3,*^, Zhang Zhang^1,2,3,*^, Yiming Bao^1,2,3,*^

**CNCB–NGDC SubCenters** (Listed in alphabetical order by database names)

**NGDC-BDV:** Xuemei Lu^3,9,47,48^, Yanan Wang^9,47,48^

**NGDC-TCM:** Yuan Yuan^49,*^, Wei Liu^49^

**NGDC-TGD:** Jinyan Huang^50^

**NGDC-NSM:** Yanfang Jiang^51,52,53^, Guoyue Lv^51,52^

**NGDC-MET:** Xinyu Liu^3,54,55^, Guowang Xu^3,54,55^

**NGDC-MOG:** Xumin Wang^56^, JiangYong Qu^56^

**NGDC-PMO:** Baisheng Li^57,58,59^, Chang Zhang^57,58,59^

**NGDC-GMG:** Xiaofeng Zou^60^, Guoxi Zhang^60^, You Guo^61^

**CNCB–NGDC PARTNERS** (Listed in alphabetical order by database names)

**Animal-APA:** Weiwei Jin^62^, Jing Gong^62^

**Animal-eRNA:** Weiwei Jin^62^, Jing Gong^62^

**Animal-SNPAtlas:** Xiaohui Niu^62^, Jing Gong^62^

**AnimalTFDB:** Wenkang Shen^63^, Anyuan Guo^63^

**BBCancer:** Zhixiang Zuo^64^, Jian Ren^64^

**CancerSEA:** Xinxin Zhang^65^, Yun Xiao^65^, Xia Li^65^

**CellMarker:** Xinxin Zhang^65^, Yun Xiao^65^, Xia Li^65^

**CGDB:** Dan Liu^66^, Yu Xue^66^

**CGGA**: Zheng Zhao^67^, Tao Jiang^67^

**circAtlas:** Wanying Wu^68^, Fangqing Zhao^3,68,69^, Jinyang Zhang^68^

**CirFunBase**: Xianwen Meng^70^, Ming Chen^70^

**ConsRM:** Bowen Song^71^, Jia Meng^72^

**CPLM:** Yujie Gou^66^, Miaomiao Chen^66^

**dbPSP & THANATOS:** Di Peng^66^, Yu Xue^66^

**DEG & DoriC:** Hao Luo^73,74,75^, Feng Gao^73,74,75^

**DirectRMDB:** Jie Jiang^71,72^, Kunqi Chen^76,77^

**DrLLPS:** Xinhe Huang^66^, Yu Xue^66^

**eLMSG:** Wan Liu^5^, Guoqing Zhang^5,6^

**EPSD:** Chi Zhang^66^, Yu Xue^66^

**EVAtlas:** Chunjie Liu^63^, Anyuan Guo^63^

**EVmiRNA:** Guiyan Xie^63^, Anyuan Guo^63^

**GenTree:** Hao Yuan^3,78^, Tianhan Su^3,78^, Yong E. Zhang^3,78^

**GTDB:** Chenfen Zhou^5^, Guoqing Zhang^5,6^

**HCL**: Yincong Zhou^70^, Ming Chen^70^, Guoji Guo^79^

**hTFtarget:** Qiong Zhang^63^, Anyuan Guo^63^

**iEKPD:** Shanshan Fu^66^, Miaoying Zhao^66^

**IMP:** Tong Chen^80^, Yuan Yuan^49^

**iPCD:** Dachao Tang^66^, Yu Xue^66^

**iUUCD:** Ming Lei^66^, Yu Xue^66^

**LeukemiaDB:** Mei Luo^63^, Anyuan Guo^63^

**lnCAR:** Yubin Xie^64^, Jian Ren^64^

**lncRNASNP2:** Yaru Miao^63^, Anyuan Guo^63^

**lncRNASNP3**: Anyuan Guo^63^, Jing Gong^62^

**m5C-Atlas:** Jiongming Ma^76^, Kunqi Chen^76^

**m6A-Atlas:** Haokai Ye^71,72^, Kunqi Chen^76^

**m6A-TSHub:** Bowen Song^81^, Daiyun Huang^72^

**m7GHub:** Yuxin Zhang^72,81^, Bowen Song^81^

**MCA**: Yincong Zhou^70^, Ming Chen^70^, Guoji Guo^79^

**MiCroKiTS:** Di Zhang^66^, Jianzhen Peng^66^

**miRNASNP:** Chunjie Liu^63^, Anyuan Guo^63^

**msRepDB:** Xingyu Liao^82,83^, Xin Gao^82^, Jianxin Wang^83^

**ncRNA-eQTL:** Jiang Li^62^, Jing Gong^62^

**Pancan-mnvQTL:** Xiaohui Niu^62^, Jing Gong^62^

**PEA:** Guiyan Xie^63^, Anyuan Guo^63^

**PceRBase:** Chunhui Yuan^70^, Ming Chen^70^

**PlantRegMap:** Dechang Yang^84^, Feng Tian^84^, Ge Gao^84^

**Plant-ImputeDB:** Xiaohui Niu^62^, Jing Gong^62^

**PncStres:** Wenyi Wu^70^, Ming Chen^70^

**PTMD:** Cheng Han^66^, Yu Xue^66^

**RhesusBase:** Juntian Qi^85^, Ni A. An^85^, Chuan-Yun Li^85^

**RMDisease:** Xuan Wang^72^, Zhen Wei^72,86^

**RMVar:** XiaoTong Luo^64^, Jian Ren^64^

**ScRAPdb:** Jiaxing Yue^87^, Zepu Miao^87^

**SEECancer:** Xinxin Zhang^65^, Yun Xiao^65^, Xia Li^65^

**SEGreg:** Qing Tang^63^, Anyuan Guo^63^

**SNP2APA:** Anyuan Guo^63^, Jing Gong^62^

**THANATOS:** Zihao Feng^66^, Yu Xue^66^

**VFDB:** Bo Liu^88^, Jian Yang^88^

**WERAM:** Chenyu Yang^66^, Leming Xiao^66^

**ZCURVE_CoVdb:** Hao Luo^73,74,75^, Feng Gao^73,74,75^

1. National Genomics Data Center, China National Center for Bioinformation, Beijing 100 101, China.

2. Beijing Institute of Genomics, Chinese Academy of Sciences, Beijing 100 101, China.

3. University of Chinese Academy of Sciences, Beijing 100 049, China.

4. Key Laboratory of Epigenetic Regulation and Intervention, Institute of Biophysics, Chinese Academy of Sciences, Beijing 100 101, China.

5. National Genomics Data Center & Bio-Med Big Data Center, Shanghai Institute of Nutrition and Health, University of the Chinese Academy of Sciences, Chinese Academy of Sciences, Shanghai 200 031, China.

6. Shanghai Sixth People’s Hospital, Shanghai 200 233, China.

7. Guangzhou Laboratory, Guangzhou 510 005, China.

8. Hangzhou Institute for Advanced Study, University of Chinese Academy of Sciences, Hangzhou 310 024, China.

9. National Key Laboratory of Genetic Evolution and Animal Model, Kunming Institute of Zoology, Chinese Academy of Sciences, Kunming 650 201, China.

10. Yunnan Key Laboratory of Integrative Anthropology, Kunming 650 201, China.

11. National Laboratory of Biomacromolecules, CAS Center for Excellence in Biomacromolecules, Institute of Biophysics, Chinese Academy of Sciences, Beijing 100 101, China.

12. Shandong First Medical University & Shandong Academy of Medical Sciences, Taian 271 016, China.

13. China National Center for Bioinformation, Beijing 100 101, China.

14. School of Advanced Interdisciplinary Science, University of Chinese Academy of Sciences, Beijing 101 408, China.

15. School of Life Science and Bio-Pharmaceutics, Shenyang Pharmaceutical University, Shenyang 110 016, China.

16. Key Laboratory for Molecular Enzymology and Engineering of Ministry of Education, School of Life Sciences, Jilin University, Changchun 130 021, China.

17. Key Laboratory of Viral Pathogenesis & Infection Prevention and Control (Jinan University), Ministry of Education, Jinan University, Guangzhou 510 632, China.

18. State Key Laboratory of Pathogenesis, Prevention and Treatment of High Incidence Diseases in Central Asia, Clinical Medicine Institute, The First Affiliated Hospital of Xinjiang Medical University, Urumqi 830 013, China.

19. Putuo People’s Hospital, Shanghai Key Laboratory of Signaling and Disease Research, School of Life Sciences and Technology, Tongji University, Shanghai 200 092, China.

20. Department of Thoracic Surgery and Institute of Thoracic Oncology, West China Hospital of Sichuan University, Chengdu 610 041, China.

21. Western China Collaborative Innovation Center for Early Diagnosis and Multidisciplinary Therapy of Lung Cancer, Sichuan University, Chengdu 610 041, China.

22. Beijing Life Science Academy, Lutuan East Road, Beijing 102 200, China.

23. Key Laboratory of Tobacco Biological Effects, Lutuan East Road, Beijing 102 200, China.

24. Institute for Stem Cell and Regeneration, Chinese Academy of Sciences, Beijing 100 101, China.

25. College of Future Technology College, University of Chinese Academy of Sciences, Beijing 100 049, China.

26. School of Traditional Chinese Medicine, Beijing University of Chinese Medicine, Beijing 100 029, China.

27. School of Life Sciences, Beijing University of Chinese Medicine, Beijing 100 029, China.

28. Center for Excellence in Animal Evolution and Genetics, Chinese Academy of Sciences, Kunming 650 201, China.

29. Department of Physiology, School of Basic Medicine, Tongji Medical College, Huazhong University of Science and Technology, Wuhan 430 030, Hubei, China.

30. Hubei Key Laboratory of Drug Target Research and Pharmacodynamic Evaluation, Huazhong University of Science and Technology, Wuhan 430 030, Hubei, China.

31. Department of Cardiovascular Surgery, Union Hospital, Tongji Medical College, Huazhong University of Science and Technology, Wuhan 430 022, China.

32. Key Laboratory of Vascular Aging, Ministry of Education, Tongji Hospital, Tongji Medical College, Huazhong University of Science and Technology, Wuhan 430 030, Hubei, China.

33. Institute for Data-Driven Tumor Immunology, Chongqing Medical University, Chongqing 400 016, China.

34. National Clinical Laboratory on Tuberculosis, Beijing Key Laboratory for Drug-Resistant Tuberculosis Research, Beijing Chest Hospital, Capital Medical University, Beijing Tuberculosis and Thoracic Tumor Institute, Beijing 101 149, China.

35. Key Laboratory of Major Diseases in Children, Ministry of Education, National Key Discipline of Pediatrics (Capital Medical University), Beijing Key Laboratory of Pediatric Respiratory Infection Diseases, Beijing Pediatric Research Institute, Beijing Children’s Hospital, Capital Medical University, Beijing 100 020, China.

36. Sino-Danish College, University of Chinese Academy of Sciences, Beijing 100 049, China.

37. The Shanghai Tenth People’s Hospital, School of Life Sciences and Technology, Tongji University, Shanghai 200 072, China.

38. National Center, Children’s Hospital, Zhejiang University School of Medicine, National Clinical Research Center for Child Health, Hangzhou 310 052, China.

39. CAS-Key Laboratory of Synthetic Biology, CAS Center for Excellence in Molecular Plant Sciences, Chinese Academy of Sciences, Shanghai 200 032, China.

40. Zhangke (Shanghai) Jianwu Biotech Services Co., Ltd, Shanghai 200 120, China.

41. State Key Laboratory of Genetic Engineering, Fudan Microbiome Center, School of Life Sciences, Fudan University, Shanghai 200 433, China.

42. Institute of Medical Information, Chinese Academy of Medical Sciences and Peking Union Medical College, Beijing 100 020, China.

43. Department of Computer Science and Technology, Tsinghua University, Beijing 100 084, China.

44. Interdisciplinary Institute for Medical Engineering, Fuzhou University, Fuzhou 350 002, China.

45. Chinese People’s Liberation Army (PLA) Medical School, Beijing 100 853, China.

46. Department of Obstetrics and Gynecology, Seventh Medical Center of Chinese PLA General Hospital, Beijing 100 700, China.

47. Biodiversity Data Center of Kunming Institute of Zoology, Chinese Academy of Sciences, Kunming 650 201, China.

48. Yunnan Key Laboratory of Biodiversity Information, Kunming Institute of Zoology, Chinese Academy of Sciences, Kunming 650 201, China.

49. Experimental Research Center, China Academy of Chinese Medical Sciences, Beijing 100 700, China.

50. Biomedical big data center, the First Affiliated Hospital, Zhejiang University School of Medicine, Zhejiang 310 003, China.

51. Northeast Subcenter of Medical Genomics, in The First Hospital of jilin University, Changchun, China.

52. Department of Hepatobiliary and Pancreatic Surgery, General Surgery Center, First Hospital of Jilin University, Changchun, China.

53. Genetic Diagnosis Center, First Hospital of Jilin University, Changchun, China.

54. State Key Laboratory of Medical Proteomics, Dalian Institute of Chemical Physics, Chinese Academy of Sciences, Dalian 116 023, China.

55. Liaoning Province Key Laboratory of Metabolomics, Dalian 116 023, China.

56. School of Life Sciences, Yantai University, Yantai 264 005, China.

57. Guangdong Provincial Center for Disease Control and Prevention, Guangzhou 511 400, China.

58. Guangdong Provincial Key Laboratory of Pathogen Detection for Emerging Infectious Disease Response, Guangzhou 511 400, China.

59. Guangdong Workstation for Emerging Infectious Disease Control and Prevention, Chinese Academy of Medical Sciences, Guangzhou 511 400, China.

60. Institute of Urology, First Affiliated Hospital of Gannan Medical University, Ganzhou 341 000, China.

61. Medical Big Data and Bioinformatics Research Centre, First Affiliated Hospital of Gannan Medical University, Ganzhou 341 000, China.

62. Hubei Key Laboratory of Agricultural Bioinformatics, College of Informatics, Huazhong Agricultural University, Wuhan 430 070, China.

63. Department of Thoracic Surgery, West China Biomedical Big Data Center, West China Hospital, Sichuan University, Chengdu 610 041, China.

64. State Key Laboratory of Oncology in South China, Cancer Center, Collaborative Innovation Center for Cancer Medicine, School of Life Sciences, Sun Yat-sen University, Guangzhou 510 060, China.

65. College of Bioinformatics Science and Technology, Harbin Medical University, Harbin 150 081, China.

66. MOE Key Laboratory of Molecular Biophysics, Hubei Bioinformatics and Molecular Imaging Key Laboratory, College of Life Science and Technology, Huazhong University of Science and Technology, Wuhan 430 074, China.

67. Beijing Neurosurgical Institute, Capital Medical University, Beijing 100 070, China.

68. Beijing Institutes of Life Science, Chinese Academy of Sciences, Beijing 100 101, China.

69. Key Laboratory of Systems Biology, Hangzhou Institute for Advanced Study, University of Chinese Academy of Sciences, Chinese Academy of Sciences, Hangzhou 310 024, China.

70. Department of Bioinformatics, College of Life Sciences, The First Affiliated Hospital, School of Medicine, Zhejiang University, Hangzhou 310 058, China.

71. Institute of Systems, Molecular and Integrative Biology, University of Liverpool, Liverpool L69 7ZB, Liverpool, UK.

72. Department of Biological Sciences, Xi’an Jiaotong-Liverpool University, Suzhou 215 123, China.

73. Department of Physics, School of Science, Tianjin University, Tianjin 300 072, China.

74. Frontiers Science Center for Synthetic Biology and Key Laboratory of Systems Bioengineering (Ministry of Education), Tianjin University, Tianjin 300 072, China.

75. SynBio Research Platform, Collaborative Innovation Center of Chemical Science and Engineering (Tianjin), Tianjin 300 072, China.

76. Key Laboratory of Gastrointestinal Cancer (Fujian Medical University), Ministry of Education, Fuzhou, China.

77. Fujian Key Laboratory of Tumor Microbiology, Department of Medical Microbiology, School of Basic Medical Sciences, Fujian Medical University, Fuzhou 350 004, China.

78. Key Laboratory of Zoological Systematics and Evolution, Institute of Zoology, Chinese Academy of Sciences, Beijing, China.

79. Center for Stem Cell and Regenerative Medicine, Zhejiang University School of Medicine, Hangzhou, China.

80. State Key Laboratory for Quality Ensurance and Sustainable Use of Dao-di Herbs, National Resource Center for Chinese Materia Medica, China Academy of Chinese Medical Sciences, Beijing 100 000, China.

81. Department of Public Health, School of Medicine, Nanjing University of Chinese Medicine, Nanjing 210 023, China.

82. Computational Bioscience Research Center (CBRC), Computer, Electrical and Mathematical Sciences and Engineering Division, King Abdullah University of Science and Technology (KAUST), Thuwal 23 955, Saudi Arabia.

83. Hunan Provincial Key Lab on Bioinformatics, School of Computer Science and Engineering, Central South University, Changsha 410 083, China.

84. State Key Laboratory of Protein and Plant Gene Research, School of Life Sciences, Biomedical Pioneering Innovative Center (BIOPIC) & Beijing Advanced Innovation Center for Genomics (ICG), Center for Bioinformatics (CBI), Peking University, Beijing 100 871, China.

85. State Key Laboratory of Protein and Plant Gene Research, Laboratory of Bioinformatics and Genomic Medicine, Institute of Molecular Medicine, College of Future Technology, Peking University, Beijing, China.

86. Institute of Life Course and Medical Sciences, University of Liverpool, Liverpool L7 8TX, UK.

87. Sun Yat-sen University Cancer Center, Guangzhou 510 060, China.

88. NHC Key Laboratory of Systems Biology of Pathogens, Institute of Pathogen Biology, Chinese Academy of Medical Sciences & Peking Union Medical College, Beijing, China.
